# An Outbreak of Gastroenteritis Among Iranian Pilgrims of Hajj during 2011

**DOI:** 10.5812/ircmj.3681

**Published:** 2013-04-05

**Authors:** Mohammad Hassan Emamian, Golchehreh Mohammad Mohammadi

**Affiliations:** 1 Shahroud University of Medical Sciences, Shahroud, IR Iran; 2 Azzahra High School, Shahroud, IR Iran

**Keywords:** Disease Outbreaks, Gastroenteritis, Foodborne Diseases, Iran

## Abstract

**Background:**

Hajj is the largest annual mass gathering of muslims. The exorbitant crowd of pilgrims and the poor condition of personal hygiene and nutrition are the main predisposing factors for different infections and food poisonings.

**Objectives:**

This study investigates a gastroenteritis outbreak among an Iranian caravan of hajj pilgrims.

**Materials and Methods:**

Studying the outbreak was carried out through a cohort study and it investigated the attack rates of the two groups of pilgrims who had consumed or had not consumed various food products. The relative risk and 95% confidence interval of each food product was then calculated.

**Results:**

Only canned rice and fish had a relative risk higher than one, although both were not statistically significant. Therefore, it could be stated that the consumed foodstuffs have not caused this outbreak.

**Conclusions:**

None of the foodstuffs distributed among the pilgrims were the reason for this disease, and studying outbreaks in caravans with higher sample sizes is suggested.

## 1. Background

Each year, more than two million Muslims from around 140 countries depart for hajj as the largest mass gathering of the world (1). Their presence in holy places, particularly in Mecca and Medina, and the crowd of hajjis during particular times in specific locations increases the possibility of communicable disease transmission. Ethnic, hygienic and cultural differences, the amount of access to health, treatment and welfare services are among important factors contributing to the spread of contagious diseases (1). Among crowded places is the small extent of Mina, to which all hajjis should attend for several days, and perform stoning on Jamaraat sites which itself causes a bigger crowd (2). Scarcity of lavatories and their poor hygiene, accumulation of different kinds of waste materials around the tents, impossibility of providing dinettes, impossibility of using refrigerators, warm weather, etc. are among predisposing factors of disease transmission, particularly respiratory infections and foodborne disease outbreaks. Most studies considering hajj are on respiratory infections, influenza and meningitis, while other communicable and non-communicable diseases are less considered. Although diarrheal is common among hajjis, no study has documented their incidence and etiological factors (2). During the hajj of 1986, gastroenteritis has been the most common reason of hospital admissions (3). A previous study carried out during 2002 indicated diarrhea as the third reason of hospital admissions among hajjis (3, 4).

## 2. Objectives

This survey has studied the outbreak of gastroenteritis in female pilgrims of one of the Iranian hajj caravans; the results of which is especially beneficial for preventing such diseases among Iranian hajjis.

## 3. Materials and Methods

Since the pilgrims of every caravan are readily accessible, and all treatment and health measures of the caravan members are performed under the supervision of the caravan physician, studying the outbreak was performed through a cohort study. A case of gastroenteritis is defined when an individual experiences ≥ 3 loose stools, or vomiting within 24 hours (5). There were no pilgrims with cancer of the bowel, irritable bowel syndromes and other diseases that may cause symptoms similar to the above definition. Using a data collection form, the history of all female pilgrims’ exposure to different foodstuffs including bread, cheese, honey, halva, rice, canned tuna, milk, orange, pear, yogurt, cucumber, non bottled water as well as their attendance to the Khif mosque in last 36 hours was questioned. The mentioned foodstuffs were all food products used by the pilgrims during the study period. According to the occurrence of gastroenteritis, the attack rate for each foodstuff was measured using the STATA software, following by the calculation of the relative risk, risk difference and the 95% confidence interval of the relative risk.

## 4. Results

**Figure 1.**
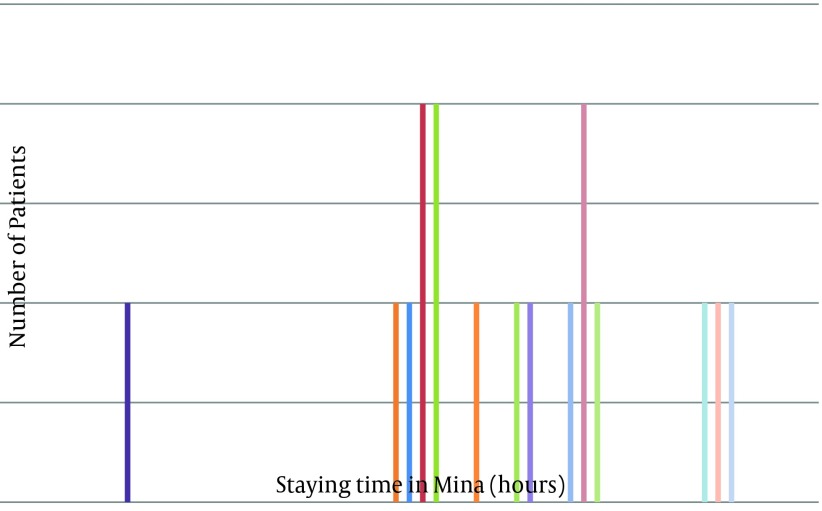
The Number of Gastroenteritis Patients According to Time of Occurrence, Hajj 2011

Among the 81 female pilgrims, 17 (attack rate=21%) suffered from gastroenteritis. The predominant symptoms were nausea, vomiting and diarrhea. The manifestation in all patients was vomiting without fever, probably due to Staphylococcus aureus, Clostridium perfringens or Bacillus cereus (6). Figure 1 indicates the outbreak curve as a common source. While the mean age of healthy women was 55.9, the mean age of patients was 54.4 years. The t-test demonstrated no significant difference between the two means.

Table 1 illustrates attack rates (AR) of each foodstuff in the two groups; those who have or have not eaten any food. The group who had eaten food had a higher AR only for canned rice and tuna, compared to those who had not eaten any food. Therefore, the relative risk of these foodstuffs is higher than one. Yet, according to the 95% confidence interval, this higher risk is not significant.

**Table 1.** The Risk of Various Foodstuffs in Developing Gastroenteritis in Iranian Hajj Pilgrims, 2011

Foods	Ate Food			Did not eat			RD^a^	Relative risk (95% CI)	P Value
	Ill	Total	Attack rate	Ill	Total	Attack rate			
**Yoghurt**	16	78	0.21	1	2	0.50	-0.29	0.41 (-0.99-0.40)	0.38
**Cucumber**	15	77	0.19	2	3	0.67	-0.47	0.29 (0.12-0.73)	0.11
**Bread**	15	77	0.19	2	3	0.67	-0.47	0.29 (0.12-0.73)	0.11
**Pears**	11	63	0.17	6	17	0.35	-0.18	0.49 (0.21-1.14)	0.18
**Orange**	15	74	0.20	2	6	0.33	-0.13	0.61 (0.18-2.06)	0.60
**Honey**	13	70	0.19	4	10	0.40	-0.21	0.46 (0.19-1.15)	0.21
**Cheese**	14	69	0.20	3	11	0.27	-0.07	0.74 (0.25-2.17)	0.69
**Spreads**	9	53	0.17	8	27	0.30	-0.13	0.57 (0.25-1.32)	0.25
**Milk**	11	63	0.17	6	17	0.35	-0.18	0.49 (021-1.14)	0.18
**Bread**	11	61	0.18	6	19	0.32	-0.14	0.57 (0.24-1.34)	0.22
**KhifMQ**	10	49	0.20	7	31	0.23	-0.02	0.90 (0.38-2.12)	1.00
**No bottled water**	5	29	0.17	12	51	0.24	-0.06	0.73 (0.29-1.87)	0.58
**Rice**	15	69	0.22	2	11	0.18	0.04	1.20 (0.32-4.53)	1.00
**Tuna conserve**	15	67	0.22	2	13	0.15	0.07	1.46 (0.38-5.62)	0.72

^a^Abbreviation: RD, Risk Differences

## 5. Discussion

Regarding Table 1, it is clear that none of the consumed food products have been the reason for the outbreak. In fact, except canned rice and tuna, other food intakes have had a protective role. The outbreak occurred among female pilgrims and the fact that only women suffered from the disease may indicate the health of the foodstuff. However the infected foodstuff might have only been distributed among women. Therefore, carrying out this study was necessary to find precise answers.

Regarding the aforementioned factors, other situations including poor hygiene of lavatories, unsafe water used for tea, votive food distribution among women (in which term, all affected patients confirmed that this had not occured), and poor environmental health in Mina might have caused infection transmission among women. Among these factors, the most likely to have caused the disease in women is using unsafe water for tea intake. The water needed for the preparation of tea in Mina is boiled by non-Iranian workers in large tanks of 1000 to 1500 liters. It has occasionally been observed that the tank water is used before reaching boiling point and using a contaminated teakettle might be the reason for the outbreak among a group of women.

As far as we know no similar study has been carried out on gastroenteritis outbreaks in hajj. Other studies demonstrate the prevalence of gastroenteritis to be 2.52 % in 2004, and 2.64 % in 2005 among Iranian hajjis (7, 8). However, it should be noted that these studies were performed during the cold months of hajj when less diarrheal diseases would occur. On the other hand, caravan physicians might have not recorded all cases during the trip. In the same article, 70 cases of gastroenteritis have been reported to have occurred in one Iranian caravan during year 2004 of hajj (8).

The following recommendations are suggested in order to prevent similar events:

Not to distribute meat containing foods and canned tuna in Mina and Arafat, regarding the increasing weather temperature in the coming years.

Boiling canned tuna and other preserved food products for 20 minutes.

Serious consideration of environmental health and waste material elimination in Mina.

Preventing entrance of non-Iranians to Iranian tent regions and the use of the same lavatories.

Maintaining the environmental hygiene of tents.

Health education and taking benefit from caravan managers and clergymen beside physicians.

Supervising boiled water provision and distribution.

Providing sufficient liquid soap in lavatories.

Keeping yogurt, cheese and other food products in the refrigerator in an appropriate temperature.

Cohort cases availability and precise record of patients and their symptoms, together with appropriate methodology were some of relative strengths of this study. However, lack of laboratory data is one important limitation. Establishing an appropriate surveillance and performing larger studies among all Iranian pilgrims (around 100.000 ones) would certainly demonstrate more precise results. This is strongly suggested due to the increase in temperature and the possibility of these kinds of outbreaks in the coming years.

In conclusion all foodstuffs distributed among pilgrims were safe and using contaminated tea or teakettle might be the reason for the outbreak.
